# Multiple determinants of lifespan memory differences

**DOI:** 10.1038/srep32527

**Published:** 2016-09-07

**Authors:** Richard N. Henson, Karen L. Campbell, Simon W. Davis, Jason R. Taylor, Tina Emery, Sharon Erzinclioglu, Cam-CAN, Lorraine K. Tyler, Carol Brayne, Edward T. Bullmore, Andrew C. Calder, Rhodri Cusack, Tim Dalgleish, John Duncan, Fiona E. Matthews, William D. Marslen-Wilson, James B. Rowe, Meredith A. Shafto, Teresa Cheung, Linda Geerligs, Anna McCarrey, Abdur Mustafa, Darren Price, David Samu, Matthias Treder, Kamen A. Tsvetanov, Janna van Belle, Nitin Williams, Lauren Bates, Andrew Gadie, Sofia Gerbase, Stanimira Georgieva, Claire Hanley, Beth Parkin, David Troy, Tibor Auer, Marta Correia, Lu Gao, Emma Green, Rafael Henriques, Jodie Allen, Gillian Amery, Liana Amunts, Anne Barcroft, Amanda Castle, Cheryl Dias, Jonathan Dowrick, Melissa Fair, Hayley Fisher, Anna Goulding, Adarsh Grewa, Geoff Hale, Andrew Hilton, Frances Johnson, Patricia Johnston, Thea Kavanagh-Williamson, Magdalena Kwasniewska, Alison McMinn, Kim Norman, Jessica Penrose, Fiona Roby, Diane Rowland, John Sargeant, Maggie Squire, Beth Stevens, Aldabra Stoddart, Cheryl Stone, Tracy Thompson, Ozlem Yazlik, Dan Barnes, Marie Dixon, Jaya Hillman, Joanne Mitchell, Laura Villis, Rogier A. Kievit

**Affiliations:** 1 Medical Research Council, Cognition and Brain Sciences Unit, Cambridge, UK; 2 Cambridge Centre for Ageing and Neuroscience, University of Cambridge and MRC Cognition and Brain Sciences Unit, Cambridge, USA; 3 Department of Psychology, Harvard University, USA; 4 Department of Psychology & Neuroscience, Duke University, USA; 5 School of Biological Sciences, University of Manchester, UK

## Abstract

Memory problems are among the most common complaints as people grow older. Using structural equation modeling of commensurate scores of anterograde memory from a large (N = 315), population-derived sample (www.cam-can.org), we provide evidence for three memory factors that are supported by distinct brain regions and show differential sensitivity to age. Associative memory and item memory are dramatically affected by age, even after adjusting for education level and fluid intelligence, whereas visual priming is not. Associative memory and item memory are differentially affected by emotional valence, and the age-related decline in associative memory is faster for negative than for positive or neutral stimuli. Gray-matter volume in the hippocampus, parahippocampus and fusiform cortex, and a white-matter index for the fornix, uncinate fasciculus and inferior longitudinal fasciculus, show differential contributions to the three memory factors. Together, these data demonstrate the extent to which differential ageing of the brain leads to differential patterns of memory loss.

Human memory is often claimed to consist of different systems, supported by distinct brain regions^1^,^2^,^3^. However, the precise fractionation of these memory systems is a matter of continuing debate^4^,^5^. One important aspect of this debate concerns whether memory systems are differentially affected in old age^6^,^7^,^8^. For example, older people often perform worse on tests of associative memory, when a new association must be made between two or more items, than they do on tests of memory for the items themselves^9^,^10^,^11^. Furthermore, the age-related impairments on these “explicit” tests of memory are typically larger than on “implicit” tests like priming, which measure effects of prior experience on current performance without making direct reference to that experience^12^,^13^,^14^.

However, much of the literature on the effects of age on memory is limited by categorical comparisons of small (N~20) groups of young (e.g., 20–30 years) versus older (e.g., 60–70 years) volunteers, where the older group often reflects self-selected and atypically-healthy/motivated individuals. Here we report data from over 300 individuals spread uniformly across the adult lifespan (18–88 years), who were recruited using more inclusive, opt-out procedures as part of the Cam-CAN cohort^15^ (www.cam-can.org). Most importantly, samples of this size also enable the comparison of different measurement (factor) models, in terms of their fit to the covariance across individuals between multiple memory measures, going beyond simple statistical tests of differences in mean scores across age groups. Here, for example, we compare various single and multi-factor models of memory that correspond to the main competing hypotheses in the literature, such as those that distinguish associative versus item memory, or explicit versus implicit memory. Moreover, the structural equation modelling (SEM) approach adopted here provides a framework to explore brain-behaviour relationships^16^, which allowed the memory factors to be related to differences in gray- and white-matter, derived from the multiple Magnetic Resonance (MR) images within the Cam-CAN cohort^17^.

Previous work examining the effects of age on memory within the SEM framework has typically relied on standardised, neuropsychological tests of memory^18^,^19^. However, these standardised memory tests are not always optimal for addressing key theoretical distinctions in memory research, such as differences between associative memory, item memory and priming. Moreover, the tests normally differ in numerous ways other than their memory demands, so while it is informative to find factors common to these memory tests (relative to tests of reasoning or language, etc.), procedural differences between them limit the ability to interpret different factors across them. Furthermore, previous tests of different types of memory typically use incommensurate behavioural measures. For example, priming is often measured in terms of (reductions in) reaction time, whereas associative and item memory are typically measured in terms of accuracy^20^. Such diverse measures potentially confound previous dissociations in the effect of age on, for example, explicit versus implicit memory. We therefore developed a single memory paradigm specifically designed to measure associative memory, item memory and priming, in which these measures 1) were extracted from the same trials, thereby closely matching non-mnemonic contributions to performance, and 2) provided a common measure of accuracy, namely discriminability according to signal-detection theory.

Another factor that is known to moderate memory is the emotionality of the material being remembered. Emotional material tends to be better remembered than neutral material, particularly for associative memory^21^,^22^, but the valence of that emotional material appears to interact with age, with older adults showing a bias towards positive relative to negative information^23^. We therefore additionally explored the effects of positive, neutral and negative valence on our measures of associative memory, item memory and priming. More specifically, participants were initially shown pictures of neutral, everyday objects superimposed on positive, negative, or neutral background scenes, and asked to relate the object and background (so-called “deep, incidental encoding”^24^). Later they were given a surprise memory test in which each trial sequentially probed their ability to: 1) name a visually-degraded version of an object (to assess priming), 2) judge whether they had seen the object in the previous phase (to assess item memory) and 3) judge whether that object had previously been paired with a positive, neutral or negative background (to assess associative memory). By relating scores on these tests to other variables like age, education-level and fluid intelligence, we were able to construct a three-factor model of memory that fit the data well, and extended previous theoretical accounts of the effect of age and emotional valence on memory. Furthermore, we were able to relate these memory factors to age-related differences gray- and white-matter estimates in a priori regions-of-interest, namely regions within the medial and ventral temporal lobes (hippocampus, parahippocampal gyrus and fusiform gyrus), which have previously been associated with different types of memory^2^,^4^, and the major white-matter tracts to/from these regions (the fornix, uncinate fasciculus and inferior longitudinal fasciculus). Only by better understanding the functional and neural bases of different types of memory can we develop future strategies to help with age-related memory loss.

## Methods

### Participants

The memory task was run on 330 of the CC700 Cam-CAN sample^15^. Five did not complete the memory task, and 11 did not complete the Cattell task (one completing neither), leaving N = 315 with complete behavioural data. An additional 10 had problems in analysis of MR images^17^. Demographic data for the remaining 305 are shown Supplementary Table 1.

The methods were carried out in accordance with guidelines approved by the Cambridgeshire 2 (now East of England—Cambridge Central) Research Ethics Committee, who approved all experimental protocols. All participants gave full, informed, written consent.

### Materials

The stimuli were taken from^25^, consisting of 160 pictures of everyday neutral objects on a yellow, square background, plus 120 pictures from the IAPS database^26^, which were grouped into 40 per valence. These 120 were selected from a larger set of 200 as the most consistently-rated in a pilot study where 12 young and 7 older participants rated the valence of each picture (ratings were very similar across age). The mean IAPS valence ratings (from 1–9, where 9 is most positive) were 7.58, 5.39 and 2.16 for positive, neutral and negative pictures respectively, while the mean arousal ratings (1–9, where 9 is most arousing) were 5.03, 3.72 and 5.99 respectively. For both valence and arousal, every mean was significantly different from every other mean, *t*’s(78) > 5.04, *p* < 3.6e-6. The number of study trials, as well as the study trial duration, was titrated in further pilot experiments, in order to ensure associative memory performance was off floor in older individuals, while item memory performance remained off ceiling in younger individuals.

The order of trials of each condition was randomized once, and the same order used for all participants in order to maximize the proportion of variance attributable to individual differences. In order to control for stimulus effects however, the 160 objects were randomly split into 4 sets, and the assignment of object sets to scene valence was rotated across participants using 4 different counterbalances.

For the priming portion of each test trial, the objects were masked by changing a random 85% of pixels to have an intensity value of 0.5 for each of the three primary colours (i.e., turned to gray). This noise level was titrated during piloting so that correct identification of an object that had not been studied before was approximately 50% (in the full Cam-CAN sample tested here, the mean rate for unstudied items was actually 44%).

### Procedure

**Figure 1:**
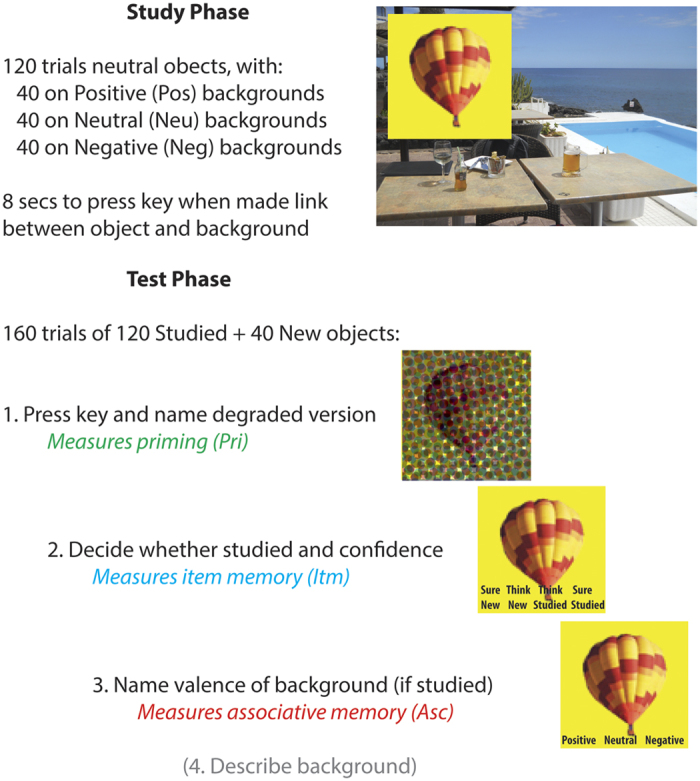
Summary of Study and Test phases.

A summary of the procedure is shown in Fig. 1. The Study phase contained 120 trials, split into 2 blocks of 10mins each, with a short break between blocks. Each Study trial began with the background scene presented for 2 seconds, after which the object was superimposed for 7.5 s, either left or right and slightly above centre. Participants were asked to press a key when they had made a story in their mind that linked the object to the scene, and continue to elaborate that story until the scene and object disappeared. There was then a 0.5s blank screen before the next trial began. Participants were informed that some scenes would be pleasant or unpleasant, but not informed that their memory would be tested later. They were given a practice session of 6 Study trials (with stimuli not used in the main experiment).

The Test phase was run after a further break of approximately 10 mins, during which refreshment was given while the experimenter chatted to the participant. This phase contained 160 trials (using 120 objects from the Study phase and 40 new objects), split into 4 blocks of approximately 20 mins each. Each Test trial began with a measure of priming: the masked version of an object appeared in the centre of the screen and participants attempted to identify the object, pressing a key and simultaneously naming it aloud, or saying “don’t know”. The pixel-noise was then removed, and item memory was tested by asking participants indicate whether the object had appeared in the Study phase and whether they were confident this was the case, by pressing one of four keys: “sure new”, “think new”, “think studied”, “sure studied” (they were told approximately ¼ of objects were new). If participants indicated “studied” (“think” or “sure”), then associative memory was tested by asking participants to say aloud whether the object had been paired with a positive, neutral, or negative background during Study (or “don’t know” if they were unable to guess). Finally, they were asked to verbally describe that scene (these qualitative data are not reported here).

#### Memory Accuracy

The proportion of valid trials and mean RTs during Study and Test are shown in Supplementary Table 2. Accuracy in the Test phase was scored with the *d*′ measure of discriminability from signal-detection theory^27^, which is the difference in inverse normal transformed probabilities of Hits (*pH*) and False Alarms (*pFA*), where *d*′ = 0 for chance performance (extreme values of 0 or 1 for *pH* and *pFA* were adjusted using an empirical log-linear approach). For associative memory, False Alarms for, e.g., Negative backgrounds, corresponded to Positive or Neutral backgrounds incorrectly recalled as “Negative”. For Item memory, Hits and False Alarms were collapsed across “sure” and “think” confidence levels (the number of low confidence judgments was too small for separate analysis). For priming, False Alarms correspond to new items correctly identified^28^. The proportions of Hits and False Alarms is shown in Supplementary Table 3, and covariances of *d*′ scores are shown in Supplementary Table 4.

#### Other Behavioural Measures

Demographic data collected from the Cam-CAN cohort include age, sex, and a self-reported measure of highest level of UK education obtained (see Supplementary Table 1), scored from 1 = ‘Basic’ (e.g., left education before 16), 2 = ‘GCSE/O-level’ (e.g., left education before 18), 3 = ‘A-level’ (e.g., left education after 18) and 4 = ‘Degree’ (e.g., left university after 21 or older). One missing education score was replaced with the mode for that age (68 years, education level 4). Fluid intelligence was measured by the Cattell Culture Fair Test14 (Scale 2, Form A), administered using pencil-and-paper according to the standard protocol, and yielding four summary sub-test scores (series completions, odd-one-out, matrices and topology). See ref. 15 for more details.

#### MRI Data

Gray-matter volume (GMV) was estimated from the combined segmentation and normalization of 1mm^3^, T1- and T2-weighted MR images; white-matter index (WMI) was estimated via fractional anisotropy (FA) from 2mm^3^, diffusion-weighted MR images (though it should be noted the relationship between FA from MR images and true white-matter health is complex and not yet fully established). For details of the MRI sequences, see ref. 15; for details of the MRI preprocessing, see ref. 17. GMV regions of interest (ROIs) were based on the prior literature and defined anatomically from the Harvard-Oxford Atlas (http://fsl.fmrib.ox.ac.uk/fsl/fslwiki/Atlases). These were: 1) hippocampus, 2) anterior parahippocampal gyrus (Brodmann Area 35, including perirhinal cortex) and 3) occipital-fusiform gyrus (Brodmann Area 37), which have previously been associated with associative memory, item memory and visual priming respectively^2^,^4^. WMI ROIs were taken from the Johns Hopkins University (JHU) atlases and included: 1) the fornix from the “ICBM-DTI-81” atlas (column, body and crescent), the 2) uncinate fasciculus and 3) inferior longitudinal fasciculus from the “tractography-20” atlas^29^. These three tracts were chosen for their connections to/from the gray-matter ROIs, and their previous association with memory^30^,^31^. All GMV and WMI measures were averaged across hemispheres. The covariances of brain measures are given in Supplementary Table 7.

### Statistics

Basic analysis of the effects of age and valence was performed by multiple regression within General Linear Models (GLMs) that treated age as a continuous variable modelled by linear and quadratic terms, and valence as a categorical variable. Age-by-Valence interactions correspond to significant effects of Valence on the linear slope of the age effect (since quadratic effects were negligible). Given the considerable sample size, we focus on effect sizes (R^2^), expressed as the percentage of variance explained by a specific statistical contrast within the GLM, rather than on significance per se, though note that R^2^ values less than 1.2% correspond to two-tailed p > 0.05. We also report adjusted R^2^ values for full GLM fit.

#### Structural Equation Models (SEMs)

SEMs were fit using the package Lavaan71^32^ in R version 3.1.2^33^. We used the following guidelines for judging good fit^34^: Root Mean Square Error of Approximation (RMSEA) below 0.05 (acceptable: 0.05–0.08) and a Comparative Fit Index (CFI) above 0.97 (acceptable: 0.95–0.97). All models were fit using Maximum Likelihood Estimation using robust standard errors, for which we report the Satorra-Bentler (SB) scaled test statistic. When models were nested, they were compared via the scaled χ^2^-difference test (note this is not simply the difference in χ^2^ for each model^35^); otherwise, non-nested models were compared via a likelihood ratio test^36^ and the Akaike Information Criteria (AIC). The significance of individual paths was tested with p-values less than 0.05 (and Z-values reported).

## Results

### Mean memory performance as function of Age and Valence

#### Main Effect of Age

**Figure 2:**
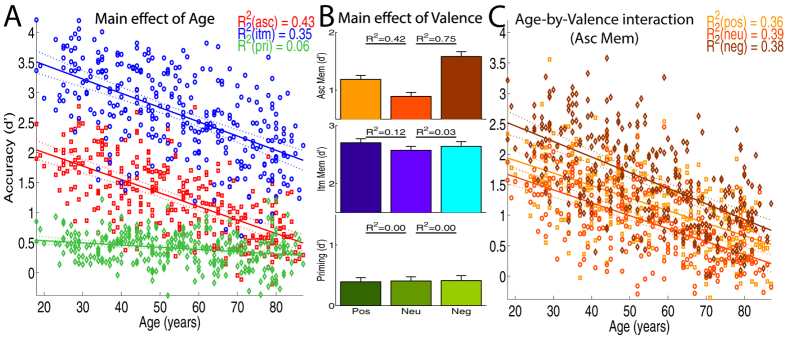
Main Effects of Age (Panel A) and of Valence (**B**) on mean accuracy for the three memory measures (Asc = Associative, Itm = Item, Pri = Priming), and their interaction for the measure of Associative Memory (Panel C; Pos = Positive; Neu = Neutral, Neg = Negative). Error bars are 95% confidence intervals.

Using a commensurate *d*′ measure of accuracy, regression analysis showed that, collapsing across valence, associative memory, item memory and priming all declined linearly with age (Fig. 2A), with no quadratic components reaching significance (R^2^ < 1%, adjusted R^2^ > 70% for full models). Associative memory declined dramatically with age (linear slope = −0.41, R^2^ = 43%), with the oldest performing only a third as well as the youngest. Item memory was better on average, but showed a comparable decline with age (slope = −0.41, R^2^ = 35%). Priming showed the least effect of age (slope = −0.07, R^2^ = 6%), with the slope being significantly less than for associative or item memory (interaction R^2^ > 27%).

#### Main Effect of Valence

The main effect of valence, collapsing across age, was examined for each type of memory separately. Associative and Item memory were better for both positive and negative trials than neutral trials (see Fig. 2B for R^2^ values for differences relative to neutral trials; full model adjusted R^2^ > 60%). Associative memory was also better for negative than positive trials (R^2^ = 12%), whereas Item memory was better for positive than negative trials (R^2^ = 3%). Thus an advantage of emotional content was seen for both associative and item memory, which was greatest for negative backgrounds when recalling their valence, but greatest for positive backgrounds when recognizing objects that were previously paired with them. Priming on the other hand showed no effects of valence (R^2^ < 1%).

#### Age-by-Valence Interactions

Finally, there was little evidence that the effects of valence interacted with age for item memory or for priming (R^2^ < 1%). Associative memory however declined faster with age for negative than for neutral or positive conditions (Fig. 2C), with valence explaining 4% of the difference in slopes in both cases (full model adjusted R^2^ > 43%). There was no evidence (R^2^ < 1%) that the slope differed for positive and neutral conditions (full model adjusted R^2^ = 42%). Thus, the advantage of negative backgrounds for associative memory was attenuated by age.

### Structural equation modelling of covariance of memory scores

The differential sensitivity of associative memory, item memory and priming to both age and emotional valence supports models in which these types of memory constitute functionally dissociable systems. However, differences in age effects could owe to a “range effect”, whereby the slope differs according to the intercept, as in a nonlinear measure that asymptotes at a lower limit for example. Moreover, such “single dissociations” between memory tests and variables like age can also be explained by a single memory system, coupled with greater measurement noise in some tests than others^8^,^37^. These are some of the issues addressed by turning to structural equation modelling (SEM), which fits the pattern of covariance between our memory measures (rather than their mean values), and allows for measurement noise to be larger in some tests than others. Moreover, we can use model selection to weigh parsimony versus explanatory power of competing memory models, testing their relative degree of support in our sample.

#### Memory measurement models

**Figure 3:**
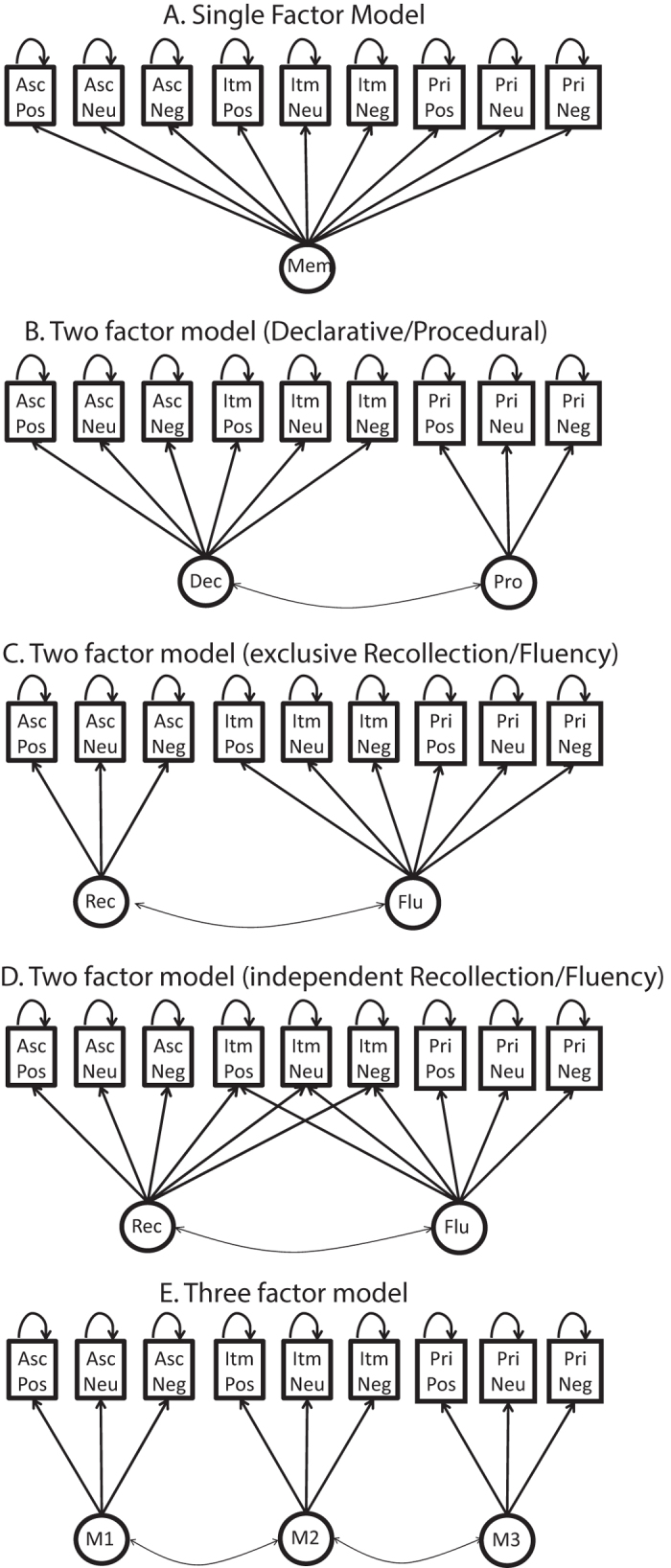
Structural Equation Models (SEMs) for the main memory theories tested, where observed variables are shown in squares (Asc = Associative, Itm = Item, Pri = Priming; Pos = Positive; Neu = Neutral, Neg = Negative) and latent variables are shown in circles (Mem = (single) Memory factor; Dec = Declarative, Pro = Procedural; Rec = Recollection, Flu = Fluency).

We used SEM to test a range of memory models from the literature. We started with a single-factor model^37^, with one memory factor driving performance on the 9 memory scores (3 valences for each of 3 memory tests; see Fig. 3). This model did not fit the data well (see Table 1 for fit statistics). A two-factor model that assumes a declarative (explicit) memory system underlying associative and item memory, plus a procedural (implicit) system underlying priming^38^, fit better than the nested single-factor model (Δχ2 = 112, Δdf = 1, p < 2.2e-16). However, an alternative, two-factor model in which a recollection factor underlies associative memory, whereas a fluency factor underlies item memory and priming (an “exclusive” model of recollection and familiarity^39^), performed better still (likelihood ratio test Z = 1.77, p = 0.039). An “independent” two-factor model in which item memory is driven by both recollection and fluency factors^24^ performed better than the nested exclusive model, despite its extra parameters (Δχ2 = 11.1, Δdf = 3, p = 0.011). However, a three factor model, in which each test is supported by a separate factor, performed better than the best two-factor model (likelihood ratio test Z = 7.45, p < 4.78e-14). The three-factor model remained the best model when equality constraints were imposed within each factor (Supplementary Table 5) and when the false alarm rate for each test was also modelled (Supplementary Table 6).

#### Effects of age and valence

**Figure 4:**
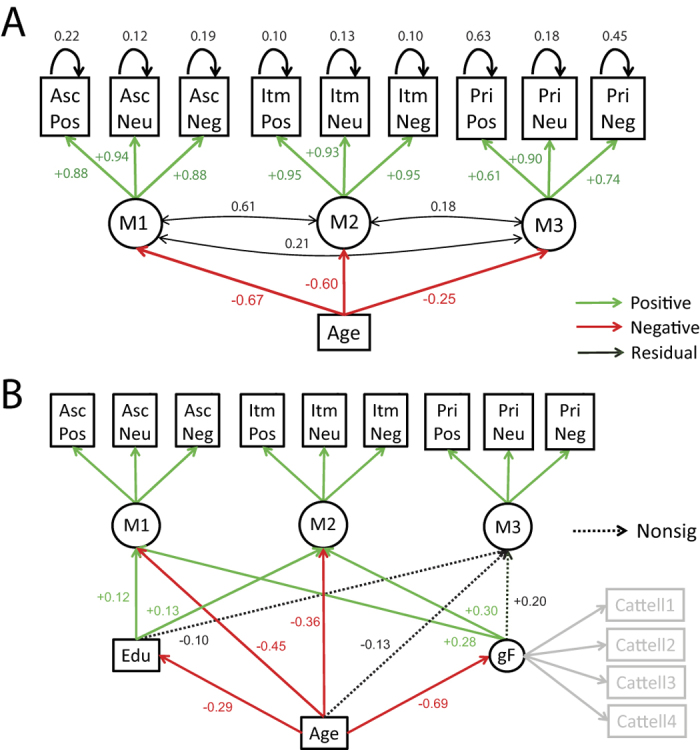
Three-factor model with Age (Panel A) and additionally with Education (Edu) and fluid intelligence (gF) (Panel B). Note that residual variances and covariances, and significant Cattell loadings, are not shown in (**B**) for sake of clarity. See Fig. 3 legend for more details.

The winning 3-factor model was then expanded to test the effects of age and valence. Adding age as a common cause of individual differences in each memory factor produced a reasonable overall model fit (Table 1), with parameters shown in Fig. 4A. As expected, owing to the relatively high measurement noise associated with priming^37^, residual variance was, on average, higher for the priming scores (0.42) than for item (0.11) or associative (0.18) memory scores. Age had the most negative influence on the M1 factor underlying associative memory (Z = −14.4), and least negative (though significant) influence on the M3 factor underlying priming (Z = −3.80). Indeed, this model was better than one in which the three age-factor paths were constrained to be equal (Δχ^2^ = 58.6, Δdf = 2, p < 1.9e-13), supporting the claim of differential effects of age on these types of memory. More focused analysis continued to show a significant reduction in model fit when equating the paths from age to M1 and M2 (while leaving the path from age to M3 free), Δχ^2^ = 4.84, Δdf = 1, p = 0.028, demonstrating differential effects of ageing on associative and item memory (and going beyond regression results in Fig. 2).

We next explored the effects of emotional valence by adding “positive” and “negative” latent factors, measured by positive and negative associative and item memory variables (but not priming, given that there was no evidence for an effect of valence on priming; see Fig. 2). These valence factors in turn depended on age (Supplementary Figure 2). The age-negative path was significant (Z = −4.48), but the age-positive path was not (Z = −0.54), supporting the conclusions from Fig. 2C that age reduces the advantage of negative backgrounds on associative memory. However, several aspects of the model fit suggested caution. Firstly, the loadings for both valence factors on both memory scores were non-significant (Z < 0.72), suggesting the shared variance due to the valence of the stimuli was modest at best. Second and most importantly, the estimated covariance matrix of the latent variables was not positive definite, suggesting model fit should be interpreted with caution. Together, these results suggest that the valence factors play, at best, a minor role in explaining covariance patterns above and beyond the memory factors (despite their strong effects on mean memory scores), leading to unstable model estimates. To avoid such issues, we dropped the valence factors from further model comparisons.

Thus far, the SEM results support a three factor memory model, where the memory factors show differential sensitivity to age. Next we expand the models by including additional demographic and neural determinants of age-related differences in memory capacity.

#### Allowing for Education Levels and Fluid Intelligence

A previous study^40^ showed that longitudinal effects of age on memory are smaller than effects of “age” estimated from cross-sectional data, owing to cohort effects related to year of birth. Nonetheless, the same authors showed that longitudinal and cross-sectional age effects become more similar when cross-sectional data are adjusted for education level (since education has generally improved over the decades). In line with this suggestion, we added educational level to our model (which did indeed increase with birth year in our cohort; Supplementary Table 1). Even though factors M1 (Z = 4.48) and M2 (Z = 4.22) underlying associative and item memory (but not M3 underlying priming, Z = −0.64) were predicted by Education, all three memory factors continued to be significantly influenced by age (Z < −3.79), suggesting that the present effects of age on memory do not reflect solely cohort differences in education.

A second question is whether these age-related declines in memory are simply a consequence of the well-known age-related decline in fluid intelligence^41^,^42^. We therefore added fluid intelligence to the above model, as a latent factor of the four Cattell sub-scores. As expected, fluid intelligence decreased significantly with age (R^2^ = 55%; Supplementary Fig. 1). Both M1 (Z = 4.28) and M2 (Z = 3.47) were affected by fluid intelligence. Crucially, despite this strong influence of fluid intelligence, the negative influence of age on M1 (Z = −6.52) and M2 (Z = −4.75) memory factors remained significant, albeit smaller in magnitude (Fig. 4B), suggesting that associative and item memory decline with age above and beyond any concomitant decline in fluid intelligence. Interestingly however, with both education and fluid intelligence in the model, the effect of age on M3, the factor underlying priming, was no longer significant (Z = −1.28).

### Dependence on brain measures

To determine whether these memory factors have different neural substrates, we extracted estimates of gray-matter volume (GMV) from the T1- and T2-weighted MR images, and white-matter index (WMI) via fractional anisotropy from the diffusion-weighted MR images. We chose three bilateral ROIs for each of GMV and WMI, based on their prior association with associative memory, item memory, and priming, as well as the integrated nature of their anatomy within the medial temporal lobe (see Methods). GMV in hippocampus, anterior parahippocampus and fusiform all decreased linearly with age (R^2^ = 28%, 18% and 41% respectively), after adjusting for total intracranial volume (TIV), with additional quadratic components for hippocampus (R^2^ = 11%) and anterior parahippocampus (R^2^ = 6%), reflecting accelerated decreases in older ages (Supplementary Figure 3). WMI in the fornix decreased dramatically with age (R^2^ = 61%), with an additional quadratic component (3%) reflecting accelerated decline in old age, whereas WMI in the inferior longitudinal fasciculus and uncinate fasciculus decreased to lesser extents (R^2^ = 26% and 7% respectively; Supplementary Figure 4), with little evidence of quadratic components (R^2^ < 1%).

**Figure 5:**
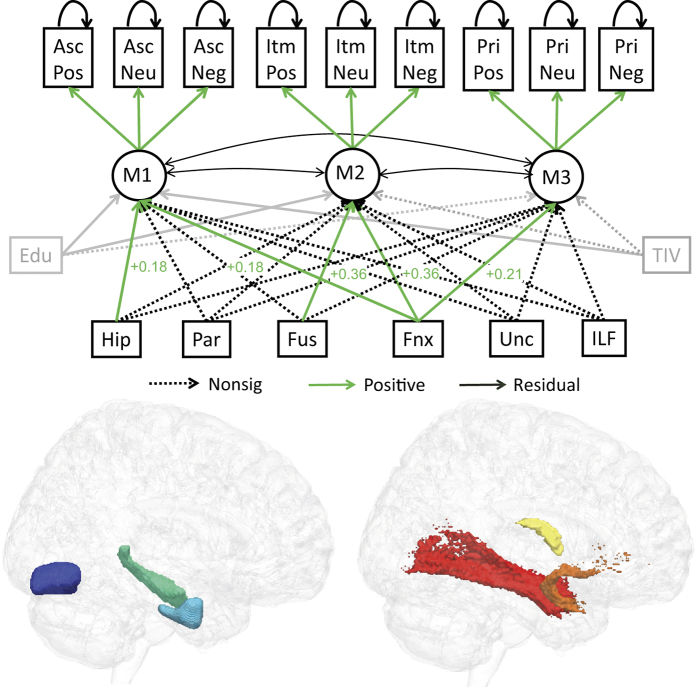
Three-factor model augmented with estimates of gray-matter volume (GMV) from Hippocampus (Hip; green), anterior parahippocampal cortex (Par, cyan) and Fusiform (Fus, blue), and a white-matter index (WMI) from Fornix (Fnx, yellow), Uncinate Fasciculus (Unc, orange) and Inferior Longitudinal Fasciculus (ILF, red), as well as Education (Edu) and Total Intracranial Volume (TIV). All brain measures are allowed to covary, but only paths from brain measures to memory factors are shown for clarity. See Fig. 3 legend for more details.

Data from these ROIs were included in a new SEM as causes of the memory factors (Fig. 5). Note that, for these models, we included Education for reasons explained previously, but did not include fluid intelligence, because our aim was to try to explain memory factors in terms of brain measures only (rather than other cognitive variables). Nonetheless, the same pattern of model comparison results remained when we did include fluid intelligence (Supplementary Table 8).

Five paths from the brain variables to memory factors were significantly positive: M1 was associated with hippocampal GMV (Z = 2.07) and fornix WMI (Z = 6.20); M2 was associated with fusiform GMV (Z = 1.99) and fornix WMI (Z = 5.99), and M3 was associated with fornix WMI (Z = 2.17). However, one should not conclude that only significant paths are important; these paths are simply those that capture unique covariance between brain variable and memory factor, beyond the (high) variance shared between the brain variables. In other words, we are not “localizing” memory factors to individual regions or tracts. However, we can use model comparison to test whether particular combinations of brain variables are important.

The first question addressed by model comparison was whether the GMV and WMI variables explained significant variance in the memory factors. Setting either the GMV or WMI paths to zero reduced model fit significantly (Δχ^2^ = 25.8, Δdf = 9, p < 2.2e-3, and Δχ^2^ = 65.4, Δdf = 9, p < 1.2e-10, respectively), demonstrating that gray matter and white matter make independent contributions to memory performance.

A second question was whether each of the GMV or WMI variables made differential contributions to the memory factors. For the GMV variables, this was tested by constraining the paths from each GMV ROI to be equal (but non-zero) across factors, while leaving the paths from the other GMV ROIs to the same factor (and all paths from the WMI tracts) as unconstrained. This significantly reduced model fit, Δχ^2^ = 25.3, Δdf = 6, p < 3.1e-4. The same was true when constraining paths from each WMI tract to be equal across factors, Δχ^2^ = 17.2, Δdf = 6, p < 8.7e-3. These results reveal another way in which the three memory factors are dissociable; in this case, in terms of their differential dependence on specific measures of brain anatomy. Furthermore, a model in which the paths from each GMV ROI were constrained to be equal for M1 and M2 (but M3 remained free) was also significantly worse, Δχ^2^ = 11.6, Δdf = 3, p < 9.0e-3, showing that the factors underlying associative and item memory have different dependencies on brain structure (though the same was not true for the WMI tracts, Δχ^2^ = 0.63, Δdf = 3, p = 0.891).

#### Effects of Age

The next question was whether these brain measures captured the effect of age on memory. Age was therefore added to the model, connected to the three memory factors. The overall fit was significantly better than a model in which age was included, but paths from age to memory factors were fixed as zero, Δχ^2^ = 52.3, Δdf = 3, p < 2.26e-11, demonstrating that there are age-related changes in memory factors that are not fully explained by our six brain measures. This is not particularly surprising, since it is likely that our structural measures may not fully capture the functionality of the regions of interest.

More interesting was the converse test, where the paths from the GMV ROIs to the memory factors were set to zero instead, such that only age and WMI could affect memory factors. This resulted in a significant drop in model fit (Δχ^2^ = 19.8, Δdf = 9, p = 0.019), demonstrating that our a priori measures of gray-matter volume explain a significant proportion of memory variance above age. The same was not true when zeroing the influence of the WMI tracts however (Δχ^2^ = 9.47, Δdf = 9, p = 0.395), demonstrating that WMI did not make a unique contribution beyond age and GMV.

Finally, it is possible that GMV and/or WMI in other ROIs capture additional memory-related variance beyond the 6 ones we chose a priori (e.g., in prefrontal cortex^43^). We therefore performed a principal component analysis (PCA) of the remaining gray- and white-matter ROIs, and found that only the first principal component of the remaining 52 bilateral gray-matter ROIs explained additional memory-related variance beyond our a priori ROIs (see Supplementary Analysis 1). Furthermore, when we added age to the SEM, this first principal component of GMV no longer explained significant memory-related variance, unlike our 3 a priori gray-matter ROIs, which continued to explain significant memory-related variance above that explained by age. These results reinforce the importance of our selected ROIs, in turn supporting the evidence in the literature that suggested them.

## Discussion

By combining multiple behavioural, demographic and brain measures from a large population-derived sample of adults from across the lifespan, we provide evidence that age-related differences in three types of human memory are both functionally and neurally dissociable. Measures of explicit memory (namely associative and item memory) decreased considerably with age from 18–88, even when allowing for educational differences across the decades and for age-related decreases in fluid intelligence, whereas a measure of implicit memory (visual priming) showed no evidence for a decline after adjusting for education and fluid intelligence. Furthermore, associative memory and item memory showed dissociable effects of the emotional valence of the stimuli, and the advantage in associative memory for negative stimuli decreased faster with age than associative memory for neutral or positive stimuli. These functional dissociations are consistent with a three-factor model of memory that was also favoured by comparison of SEM instantiations of prominent theories in the literature^24^,^37^,^38^,^39^. Further SEM modelling revealed that the three memory factors have differential sensitivity to age, as well as different contributions from gray- and white-matter in *a priori* regions of interest. Our results therefore demonstrate that age-related declines in memory are unlikely to be driven by a unitary underlying cause, such as slowing of processing speed^44^, or degradation of sensory input^45^; rather, these memory declines arise from multiple memory systems.

While claims about functional and neural dissociability of age-related memory changes have been made before^7^,^46^, there are several reasons why our findings represent important steps forward. Firstly, we used a single paradigm carefully designed to isolate three types of memory, together with a commensurate measure of performance, rather than the different memory tasks and/or different memory measures typically used. This provides much tighter control of performance differences across memory tasks, reducing non-mnemonic confounds. Moreover, our large sample enabled the use SEM to fit the covariance between memory measures, driven by individual differences, rather than simply examining differences in mean performance between groups of people. While techniques like SEM have been applied to other large datasets in previous studies, the measures of memory in these studies nearly always come from a range of diverse standardized tests. While it is certainly valuable to find common memory factors across diverse tests, the converse case of inferring different memory factors becomes more difficult when other procedural differences exist between the tests. While our three factor solution could still reflect minor procedural differences between the types of decisions required by our paradigm (e.g., identification in the case of our priming measure, versus three- or four-way decisions in the case of our associative and item measures), the fact that the measures were derived from the same trials (thereby controlling for state-dependent differences, e.g., attention or motivation), and potentially influenced each other, would only increase the correlation between measures, reducing the chance of finding dissociations among them.

Secondly, many previous studies have compared small groups of young and old volunteers responding to adverts, with potential selection bias in both groups, particularly for older adults. While it is likely that our older individuals were not truly representative – they were constrained for example by contraindications to MR scanning – our CamCAN cohort is reasonably representative of the larger UK population on demographic variables, and is certainly less biased than typical group studies, and thus more generalizable. Furthermore, by utilizing demographic variables like education, and other cognitive measures like fluid intelligence, we were able to adjust for other concomitant differences that often exist between age groups.

Thirdly, few studies addressing the multifactorial nature of memory have benefited from the combination of high-resolution T1-weighted, T2-weighted and diffusion-weighted MR images, allowing independent estimation of gray- and white-matter. Although often considered the gold standard, manual delineation of ROIs becomes prohibitive for large cohorts, so we used state-of-the-art automated techniques to jointly segment the T1 and T2 images, and to register these segments, as well as the diffusion-weighted data, to a sample-specific template, minimizing age-bias^47^. This allowed us to look at regionally-specific effects of age, and their relation to memory, going beyond previous SEM-based studies that have used only single, global measures of brain structure like Total White Matter^18^ (see also Supplementary Analysis 1).

Despite these steps forward, it is important to remember that our data are cross-sectional. This makes it difficult to distinguish true effects of “age” from cohort effects related to year of birth. Indeed, longitudinal studies^40^ have shown important differences between the effects of age and effects of birth year. In the case of memory, a lot of the variance related to birth year can be captured by differences in education, and our effects of age remained after adjusting for educational level. Nonetheless, other differences across the generations (e.g., nutrition, lifestyle, culture) could contribute to our age effects. However, even if we cannot identify the precise causes of individual differences related to birth year in our sample, our data demonstrate that these causes affect different types of memory in different ways.

What do the three factors represent in the model favoured by our SEM comparisons? While M1 and M3 may be usefully interpreted in terms of the prior theoretical constructs of “recollection” and (visual) “fluency”^2^,^3^, interpretation of M2 in terms of “familiarity” deserves caution. This is because our measure of item memory (recognition memory) is generally believed to be a function of both recollection and familiarity^24^. There are methods to separate the contributions of these two (e.g., fitting “dual-process” models to Receiver-Operating Curves, ROCs^48^), but unfortunately item memory was too close to ceiling for meaningful analysis of ROCs across our four levels of confidence. We did test a SEM version of an independent dual-process model (Table 1), in which item memory loaded on both a “recollection” factor (that also loaded on associative memory) and a “fluency” factor (that also loaded on priming), but this model was not preferred over our three-factor model. However, this “independent two-factor” model is not necessarily appropriate for theories that associate familiarity with fluency of *conceptual* processing^49^, since our tests of priming were more likely to index fluency of *perceptual* processing. It is also possible our M2 factor includes contributions of “non-criterial” recollection^24^ – i.e, memory for aspects of the Study episode in which an object was presented, other than the background with which it was paired (as required for our test of associative memory). According to dual-process theory, this would mean our M2 factor is a mixture of (non-criterial) recollection and familiarity, and if only the recollective component were affected by age, then this could reconcile our findings with a previous study arguing that age does not affect familiarity^48^. We therefore restrain from labelling our M2 factor as “familiarity”. We note also that the range of models we considered here is limited by the data provided by our paradigm, and convergent evidence about the nature of memory factors is needed from other paradigms with different experimental manipulations. Nonetheless, a more general implication of our results is that, while two-factor models may sometimes provide an adequate fit to data, a three-factor model more adequately captures the independent sources of individual differences in memory across the adult lifespan.

More importantly for the present focus on ageing is our SEM finding that the M2 factor underlying item memory showed a smaller decrease with age than our M1 factor underlying associative memory. Nonetheless, though significantly different from the loading of age on M1 (−0.67), the negative loading of age on M2 (−0.60) was still considerable. This is contrary to generic claims that age has little effect on item memory^9^,^10^,^11^, but is consistent with several studies that show that the difference between associative and item memory is reduced when encoding is incidental^50^ and/or encourages semantic associations^51^. This supports claims that older people show less of a specific deficit in memory when semantic elaboration is “supported” by the task^52^. Importantly, it does not appear to be because our tests were measuring the same “thing”: Not only did our SEM support separable factors in terms of covariance across individuals, but associative and item memory were affected differently by emotional valence (see below), and were dependent on different combinations of brain structures.

Our findings support prior claims^7^,^12^,^13^ that age exerts greater effects on explicit memory, such as associative or item memory, than on implicit memory (priming). This claim has been contested by recent modelling efforts that allow for different measurement noise in each type of test^8^,^20^. Importantly, our use of SEM allowed us to address this possibility by modelling test-specific residual variance. Our results clearly indicate that age has weaker effects on our factor (M3) underlying priming than on our two explicit memory factors, despite this greater measurement noise. Indeed, once including education and fluid intelligence, there was no longer evidence for a unique contribution of age to M3 in the SEM model. This suggests that the systems/processes underlying visual priming are age-invariant, though caution is always warranted for a null result, and larger (or longitudinal) studies may indicate a unique effect of age on perceptual priming, or on other types of implicit memory.

Associative memory was best for negative backgrounds, whereas item memory was best for objects presented on positive backgrounds. Busby and Burgess^53^ reported the opposite pattern of impaired associative memory but improved item memory for negative relative to neutral information, but an important difference is that in their study, item memory was tested for the emotional stimulus itself, while the background scene was neutral. One possible explanation of both results is that negative stimuli attract more attention at encoding^54^,^55^. Thus in our paradigm, negative scenes may attract attention towards the background and away from the foreground object, improving associative memory but impairing item memory, whereas in the Busby and Burgess paradigm, attention may have been drawn towards the negative foreground items instead (though see ref. 53 for counter-arguments). However, such an attentional account would predict an effect valence on subsequent priming (i.e, less priming when attention to objects at encoding is drawn away to negative backgrounds); for which we found no evidence. Moreover, this attentional account is not sufficient to explain why associative and item memory were better for both negative and positive backgrounds relative to neutral ones. It seems more likely that this general advantage for emotional versus neutral conditions reflects another factor, such as the fact that positive and negative backgrounds had higher arousal ratings than neutral ones (see Methods); or that negative (and positive) stimuli do benefit from a genuine advantage in memory beyond attention at encoding^53^.

More relevant to the present focus on ageing is the finding that the associative memory advantage for negative backgrounds decreased with age. This is generally consistent with the “positivity bias” hypothesis^23^,^56^. Nonetheless, the same age-related decrease was not seen for positive (relative to neutral) backgrounds, suggesting that this effect is driven primarily by a bias “away” from negative stimuli, rather than “towards” positive stimuli, consistent with several previous studies^54^,^55^,^57^. This bias is unlikely to reflect a difference in the perceived valence of the scenes, because pilot data showed no difference between groups of young and older adults in their classification of the valence of the IAPS scenes used here. Furthermore, this effect of age only appeared in associative memory (not item memory), suggesting that the age-related bias is not a general attentional effect; rather, the bias in the present data appears to have a mnemonic basis, selective to associative memory, further supporting different memory systems underlying associative and item memory.

Although all six of our GM and WM ROIs showed decreases with age, the correlation between these age-effects was not perfect, suggesting that different aspects of brain structure age differentially (see also refs 58 and 59), potentially explaining the different effects of age on the three memory factors. Indeed, we found that hippocampal GMV made a unique, positive contribution to the M1 factor. This is consistent with the hypothesized role of hippocampus in associative memory^2^,^4^, despite mixed findings in prior attempts to associate memory scores with structural brain measures^60^,^61^,^62^ (though see refs 63 and 64). However, any simple dependence of M2 on anterior parahippocampus (as expected from prior association of perirhinal cortex with familiarity^65^), was not supported; nor was any simple dependence of M3 on fusiform (as expected from prior association of this region with visual priming^66^). This suggests that there is no simple one-to-one mapping of the memory factors onto single brain regions (or at least, onto the three ROIs considered here). But this is not necessarily surprising, since some theories propose that different memory functions arise from interactions between multiple brain regions^67^. More important therefore was the finding that the three GMV ROIs made differential contributions to the three memory factors, supporting more general claims that memory systems are neurally dissociable. Moreover, the importance of these specific ROIs was reinforced by the finding that they explained unique memory-related variance beyond the first principal component of the remaining gray- and white-matter ROIs (see Supplementary Analysis 1).

In terms white-matter, we found the fornix made unique contributions to all three memory factors. Its contribution to associative memory is consistent with previous studies^30^,^68^ and it is interesting that this contribution was distinct from that of the hippocampal GMV (since both were included in same model), suggesting that tissue integrity within the hippocampus can vary independently from axonal loss in the main output of the hippocampus. This reinforces the importance of examining both gray- and white-matter measures in relation to cognitive function^42^ and multiple brain measures more generally^69^. No unique contributions were found from the uncinate or inferior longitudinal fasciculi, but again, SEM comparison allowed us to conclude more generally that the three WM measures made differential contributions to memory^19^.

When we added age to our SEMs, model comparison showed that the three GM ROIs as a whole continued to explain significant additional variance. The combined findings that 1) age is associated with reduced memory performance, 2) age is associated with reduced GMV, and that 3) GMV is associated with reduced memory performance even after allowing for age, constitute the basis of formal mediation analysis. While this might suggest that GMV mediates the age-related differences in memory performance, such an interpretation is problematic when based on cross-sectional data^70^. Nonetheless, the ultimate aim of SEMs like ours would be to explain the cognitive data fully with brain (or other biological) measures, such that chronological age (cross-sectional or longitudinal) no longer explains significant additional variability. We clearly could not achieve this goal with the present data, which is perhaps not surprising, since it is likely that other types of brain measurement (e.g., functional activity and functional connectivity), are necessary to explain the full pattern of age-related differences in memory.

In conclusion, memory is a vital human capacity, as evident in the dismay caused by memory problems as we grow older. However, memory is a multi-faceted capacity, and by better understanding which aspects of memory decline with age and which are preserved, as well as how these differences relate to changes in underlying brain structure, we can better design strategies aimed at minimizing the impact of age-related memory loss.

## Additional Information

**How to cite this article**: Henson, R. N. *et al*. Multiple determinants of lifespan memory differences. *Sci. Rep.* **6**, 32527; doi: 10.1038/srep32527 (2016).

## Supplementary Material

Supplementary Information

## Figures and Tables

**Table 1:** Fits of the main SEMs tested.

Model	χ^2^	df	RMSEA	CFI	SB	AIC
Single-factor (A), e.g., Berry *et al*.^37^	645	27	0.270	0.722	1.080	2838
Two-factor (B), e.g., Squire^38^	424	26	0.220	0.821	1.032	2580
Two-factor (C), e.g., Gardiner *et al*.^39^	340	26	0.196	0.859	1.001	2483
Two-factor (D), e.g., Yonelinas^24^	324	23	0.211	0.855	0.940	2472
Three-factor (E)	78	24	0.084	0.976	0.946	2220
						
Three Factor with Age	80	30	0.072	0.980	0.946	2928
						
Three Factor with Age & Edu	94	36	0.071	0.977	0.953	3778
						
Three Factor with Age, Edu & gF	214	78	0.074	0.957	0.977	6725
						
Three Factor with GM and WM ROIs*	118	72	0.046	0.981	0.982	7815
Three Factor with GM constrained*	142	78	0.052	0.974	0.983	7828
Three Factor with WM constrained*	135	78	0.049	0.977	0.984	7821
						
Three Factor with GM, WM and Age*	139	85	0.046	0.979	0.980	8282
Three Factor with WM, Age, GM-nulled*	159	94	0.048	0.974	0.979	8283
Three Factor with GM, Age, WM-nulled*	149	94	0.044	0.972	0.985	8273

The SEMs are grouped according to those fitting the same data (same observed variables), since one cannot compare all model fit indices across fits to different data. Edu = Education Level; gF = Fluid Intelligence; GM = Gray Matter (Volume); WM = White Matter (Index). For fit measures, see Methods (df = degrees of freedom left in data). *=These models additionally contained Education and TIV (affecting all memory factors). Note that the χ^2^ reported is the Satorra-Bentler scaled χ^2^, with the scaling factor reported as SB.
