# Supplementary material for: Multiple determinants of lifespan memory differences
Source: Sci Rep. 2016 Sep 7;6:32527. doi: 10.1038/srep32527 (PMC5013267; doi:10.1038/srep32527)

SUPPLEMENTARY INFORMATION FOR:

Multiple determinants of lifespan memory differences

Richard N. Henson 1*
Karen L. Campbell 2
Simon W. Davis 3
Jason R. Taylor 4
Tina Emery 1
Sharon Erzinclioglu 1
Cam-CAN 5
& Rogier A. Kievit 1

1 Medical Research Council, Cognition and Brain Sciences Unit, Cambridge, UK
2 Department of Psychology, Harvard University, USA
3 Department of Psychology & Neuroscience, Duke University, USA
4 School of Psychological Sciences, University of Manchester, UK
5 Cambridge Centre for Ageing and Neuroscience, University of Cambridge and MRC Cognition and Brain Sciences Unit, Cambridge, UK

*Corresponding author:
Prof. Richard Henson
MRC Cognition & Brain Sciences Unit
15 Chaucer Road
Cambridge, CB2 7EF
UNITED KINGDOM
Telephone: +44 1223 355 294
Fax: +44 1223 359 062
Email: rik.henson@mrc-cbu.cam.ac.uk

Supplementary Results

Supplementary Table 1: *Demographics of all participants with valid cognitive and brain data.*

Decade		1	2	3	4	5	6	7
N		21	47	48	50	48	48	43
**Age range (years)**		18 – 27	28 – 37	38 – 47	48 – 57	58 – 67	68 – 77	78 – 87
**Sex (male/female)**		11/10	23/24	23/25	26/24	24/24	23/25	24/19
**Highest Education**								
	4 Degree	14	40	33	35	32	18	20
	3 A-Level	4	4	9	10	7	12	10
	2 GSCE	3	3	6	4	7	9	5
	1 Basic	0	0	0	1	2	9	8

Education levels scored from 1 = ‘Basic’ (e.g., left education before 16), 2 = 'GCSE/O-level’ (e.g., left education before 18), 3 = 'A-level’ (e.g., left education after 18) and 4 = ‘Degree’ (e.g., left university after 21 or older).

Supplementary Table 2: *Proportion of valid trials and mean RTs at Study and Test.* Age is split into 7 decades, though note that in statistical analyses, it is treated as a continuous variable (see Methods). Because the distribution of valid trials over participants was negatively skewed, the mean for valid trials is shown after arcsin transformation. Reaction Times (RTs) reflect mean over participants of median RT across trials.

Decile	1	2	3	4	5	6	7
Valid Trials (median %)							
Study (Pos/40)	98	98	96	96	93	86	81
Study (Neu/40)	95	97	95	96	91	83	76
Study (Neg/40)	96	97	94	94	91	79	75
Test (/160)	100	100	100	100	100	99	99
Study RTs (s)							
Pos	3.14	2.97	3.12	3.01	3.20	3.85	4.24
Neu	3.13	3.04	3.20	3.12	3.28	3.90	4.29
Neg	3.18	3.20	3.31	3.22	3.39	3.99	4.39

The proportion of valid Study trials (in which a key was pressed within 7.5s of object onset) decreased with age (R^2=23% of linear fit against arcsin of proportion), while response times (RT) increased with age for that press (R^2=14% for linear fit), as expected given slower reactions in general with advancing age. There were also more missed trials and longer RTs for Negative than Neutral trials (R^2=3% for difference in mean proportion, and R^2=2% for difference in mean RTs), and for Neutral than Positive trials (R^2=9% and R^2=4%, respectively). However, for neither the proportion of trials, nor RTs, was there any evidence of an interaction between Age and Valence (R^2<1%).

There was a suggestion that RTs to Study objects that were later identified (named) at Test (M=3.00s) were slightly longer than trials for objects that were not identified (M=2.93s), though this was a marginal effect, R^2=1.4%. There was no evidence that Study RTs differed according to whether (M=2.99s) or not (M=3.03s) the valence of the background was recalled

(i.e, associative memory), R^2<1%. (There were too few trials in which objects were not recognized to get an accurate estimate of Study RTs for hits and misses in item memory.)

The proportion of Test trials analysed (after excluding "don't know" responses, incorrect key presses or response times less than 500ms) was too close to ceiling to analyse. Excluding trials that were missed at Study did not affect the pattern of significant results, so all Study trials were included.

Supplementary Table 3: *Hit and False Alarm Rates for each type of memory at Test.*

Decile	1	2	3	4	5	6	7
Priming							
Hit (Pos)	0.73	0.75	0.70	0.63	0.53	0.47	0.45
Hit (Neu)	0.75	0.74	0.71	0.64	0.53	0.48	0.45
Hit (Neg)	0.73	0.73	0.71	0.64	0.55	0.48	0.46
FA	0.52	0.59	0.52	0.48	0.40	0.37	0.32
Item Memory							
Hit (Pos)	0.91	0.90	0.88	0.87	0.81	0.73	0.73
Hit (Neu)	0.90	0.90	0.84	0.84	0.77	0.68	0.70
Hit (Neg)	0.92	0.90	0.86	0.85	0.79	0.72	0.69
FA	0.02	0.04	0.05	0.05	0.07	0.08	0.08
Associative Memory							
Hit (Pos)	0.61	0.60	0.59	0.54	0.51	0.47	0.48
Hit (Neu)	0.75	0.73	0.64	0.62	0.57	0.56	0.49
Hit (Neg)	0.83	0.80	0.75	0.72	0.66	0.47	0.48
FA (Pos)	0.09	0.10	0.14	0.14	0.16	0.21	0.27
FA (Neu)	0.23	0.24	0.25	0.27	0.29	0.39	0.35
FA (Neg)	0.09	0.11	0.13	0.16	0.19	0.16	0.16

Supplementary Table 4: *Covariance Matrix of* d'

	Ass Pos	Ass Neu	Ass Neg	Itm Pos	Itm Neu	Itm Neg	Pri Pos	Pri Neu	Pri Neg
Ass Pos	0.39	0.32	0.36	0.29	0.29	0.32	0.06	0.05	0.04
Ass Neu	0.32	0.38	0.39	0.28	0.31	0.32	0.06	0.05	0.04
Ass Neg	0.36	0.39	0.56	0.38	0.40	0.41	0.07	0.06	0.04
Itm Pos	0.29	0.28	0.38	0.54	0.49	0.52	0.08	0.07	0.06
Itm Neu	0.29	0.31	0.40	0.49	0.56	0.51	0.07	0.06	0.05
Itm Neg	0.32	0.32	0.41	0.52	0.51	0.60	0.07	0.05	0.04
Pri Pos	0.06	0.06	0.07	0.08	0.07	0.07	0.11	0.06	0.04
Pri Neu	0.05	0.05	0.06	0.07	0.06	0.05	0.06	0.12	0.07
Pri Neg	0.04	0.04	0.04	0.06	0.05	0.04	0.04	0.07	0.09

Supplementary Table 5: *Models with fixed factor-score loadings for each factor*

Model	χ2	df	RMSEA	CFI	SB	AIC
Single-factor (A), e.g., Berry et al. (2012)	1668	35	0.385	0.265	1.100	3960
Two-factor (B), e.g., Squire (1992)	610	33	0.236	0.740	1.004	2741
Two-factor (C), e.g., Gardiner et al. (1998)	1218	33	0.338	0.466	1.034	3389
Two-factor (D), e.g., Yonelinas (2002)	585	33	0.230	0.751	1.049	2742
Three-factor (E)	131	30	0.103	0.955	0.953	2259

Supplementary Table 6: *Models with False-Alarms included.*

In these models, the inverse normalized false alarm rate (see Methods) from new items for priming and item memory were constrained to load equally on each of the 3 corresponding d' scores (to capture any covariance induced by the shared false alarm rates subtracted from each d' score), while the corresponding false alarm rates for both "positive" and "negative" trials in the associative memory task were constrained to load equally on the 3 corresponding d' associative memory scores (note these models contain new data compared to those in the main Table 1 and Supplementary Table 5 and, so χ2 cannot be compared across these tables).

Model	χ2	df	RMSEA	CFI	SB	AIC
Single-factor (A), e.g., Berry et al. (2012)	753	59	0.193	0.772	0.952	3639
Two-factor (B), e.g., Squire (1992)	599	58	0.172	0.822	0.950	3493
Two-factor (C), e.g., Gardiner et al. (1998)	571	58	0.168	0.831	0.993	3491
Two-factor (D), e.g., Yonelinas (2002)	502	55	0.161	0.853	0.981	3422
Three-factor (E)	399	56	0.139	0.887	1.002	3328

Supplementary Table 7: *Covariance Matrix of Brain Measures*. Note that GMV was first orthogonalised with respect to TIV, and all values multiplied by 100 for display purposes. Hip = Hippocampus; Par = Anterior parahippocampal cortex; Fus = Fusiform; For = Fornix; Unc = Uncinate Fasiculus; ILF = Inferior Longitudinal Fasiculus.

	Hip	Par	Fus	For	Unc	ILF
Hip	0.33	0.21	0.19	0.17	0.06	0.04
Par	0.21	0.30	0.15	0.13	0.05	0.04
Fus	0.19	0.15	0.37	0.21	0.06	0.07
For	0.17	0.13	0.21	0.40	0.08	0.11
Unc	0.06	0.05	0.06	0.08	0.17	0.05
ILF	0.04	0.04	0.07	0.11	0.05	0.11

Supplementary Table 8: Model fits for Brain variables with fluid intelligence included

Model	χ2	df	RMSEA	CFI	SB	AIC
Three Factor with GM and WM ROIs	413	139	0.080	0.913	0.990	10784
Three Factor with GM constrained*[1]	433	145	0.081	0.908	0.990	10792
Three Factor with WM constrained*[2]	435	145	0.081	0.908	0.995	10796
Three Factor with GM, WM and Age	310	155	0.057	0.952	0.995	11114
Three Factor with WM, Age, GM-nulled*[3]	331	164	0.058	0.949	0.994	11116
Three Factor with GM, Age, WM-nulled	318	164	0.055	0.953	0.998	11105

*[1] Significant reduction in model fit relative to non-constrained, $\Delta\chi2 = 19.8$, $\Delta df = 6$, $p < .0029$

*[2] Significant reduction in model fit relative to non-constrained, $\Delta\chi2 = 21.6$, $\Delta df = 6$, $p < .0014$

*[3] Significant reduction in model fit relative to non-constrained, $\Delta\chi2 = 20.5$, $\Delta df = 9$, $p < .015$

Supplementary Analysis 1: *Principal components of other GM and WM ROIs*

To assess whether other ROIs, beyond the 3 GM and 3 WM ones that we selected a priori, contributed to memory-related variance, we performed a principal component analysis (PCA) across the remaining ROIs. For the 52 bilateral GM ROIs remaining after excluding our original 3 GM ROIs, the first 3 PCs explained 78% of the GM variance. Similarly, the first 3 PCs from a PCA across the 24 remaining bilateral WM ROIs explained 62% of the WM variance. We added these 3+3=6 PCs, together with our original 3+3 ROIs, to a SEM (together with TIV and Education, as in Figure 5 of main paper). A series of model comparisons showed that the 2nd and 3rd PCs did not improve the model fit, so these were dropped. The 1st WM PC did not improve the model fit either, $\chi 2 = 2.06$, df = 3, p = .561, but the 1st GM PC did improve the model fit (above and beyond our original 3 GM ROIs), $\chi 2 = 24.0$, df = 3, p < 2.53e-5 (i.e, zeroing the 3 paths from the 1st GM PC to the 3 memory factors worsened model fit). This demonstrates that GM outside our 3 ROIs also contributes to the three memory factors, as we elaborate in the Discussion. As a note, there was no clear clustering of ROIs in terms of their relative contributions to the first principal component of GM or WM; rather all ROIs contributed to some extent (with the same sign), making this component similar to a measure of total gray matter/total white matter.

Importantly however, the 3 GM ROIs continue to improve model fit even with the 1st PCs of GM and WM in the model, $\chi 2 = 23.8$, df = 9, p < 4.69e-3 (i.e, zeroing the 9 paths from the 3 GM ROIs to the 3 memory factors worsened model fit). Likewise, the 3 WM ROIs continue to improve model fit, $\chi 2 = 19.6$, df = 9, p < .021 (i.e, zeroing the 9 paths from the 3 WM ROIs to the 3 memory factors worsened model fit). The results demonstrate that both the GM ROIs and the WM ROIs that we selected a priori capture additional variance beyond the dominant pattern in the remaining GM and WM ROIs.

Furthermore, when we add age to the model, it continues to explain additional variance (i.e, model fit worsens when we zero 3 paths from age to memory factors, $\chi 2 = 33.7$, df = 3, p < 2.28e-7), demonstrating that even with these additional GM and WM PCs, we cannot capture all age-related change in memory. However, we also find that the 1st PC of GM no longer improves model fit, $\chi 2 = 3.83$, df = 3, p = .280 (the same applies if we zero the 3 paths from the 1st PC of WM to memory factors instead, or indeed zero all 6 paths from 1st GM and WM PCs to memory factors). This is most likely because the 1st PCs of GM and of WM correlate highly with age (R=-0.64 and R=-0.65 respectively), whereas the 3 GM and 3 WM ROIs correlate less with age on average (R=-0.42 and R=-0.52 respectively). Thus these PCs do not capture any variance in memory factors beyond that captured by age. This stands in contrast to the 3 a priori GM ROIs, which continued to improve model fit even with both age and the 1st GM and WM PCs in the model, $\chi 2 = 22.5$, df = 9, p < 7.36-e3 (the same was not true for our 3 WM ROIs, as in the main Results with additional PCs). This demonstrates that there continues to be unique variance in our

GM ROIs that explains memory variance beyond effects of age (as reported in main Results), but now even when we allow for the dominant pattern of GM across all other ROIs.

In summary, these new analyses demonstrate that there is something special about our GM and WM ROIs that is not captured by the dominant components (in sense of first 3 PCs) of the remaining ROIs, and our GM ROIs in particular continue to explain unique memory variance beyond age. Of course, our GM and WM ROIs do not explain all memory variance, nor even all age-related memory variance, with contributions of other variables like functional activity/connectivity remaining likely, as elaborated in the Discussion; but as far as our current structural brain measures are concerned, these extra analyses reinforce the importance of our selected ROIs, in turn supporting our reasons from the literature for choosing them, and highlighting them as the focus for future investigations.

Supplementary Figure 1: Fluid Intelligence as estimated from Cattell factor score against age

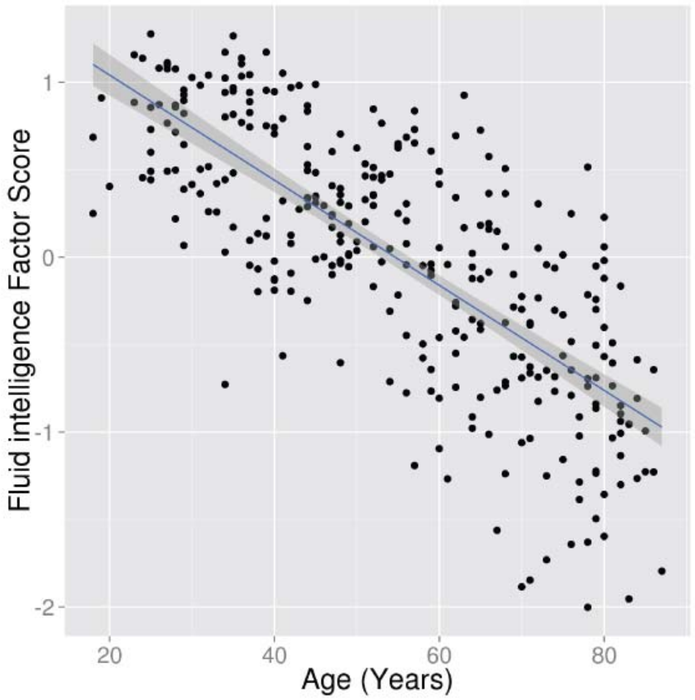

Supplementary Figure 2: SEM with Positive and Negative Valence factors included.

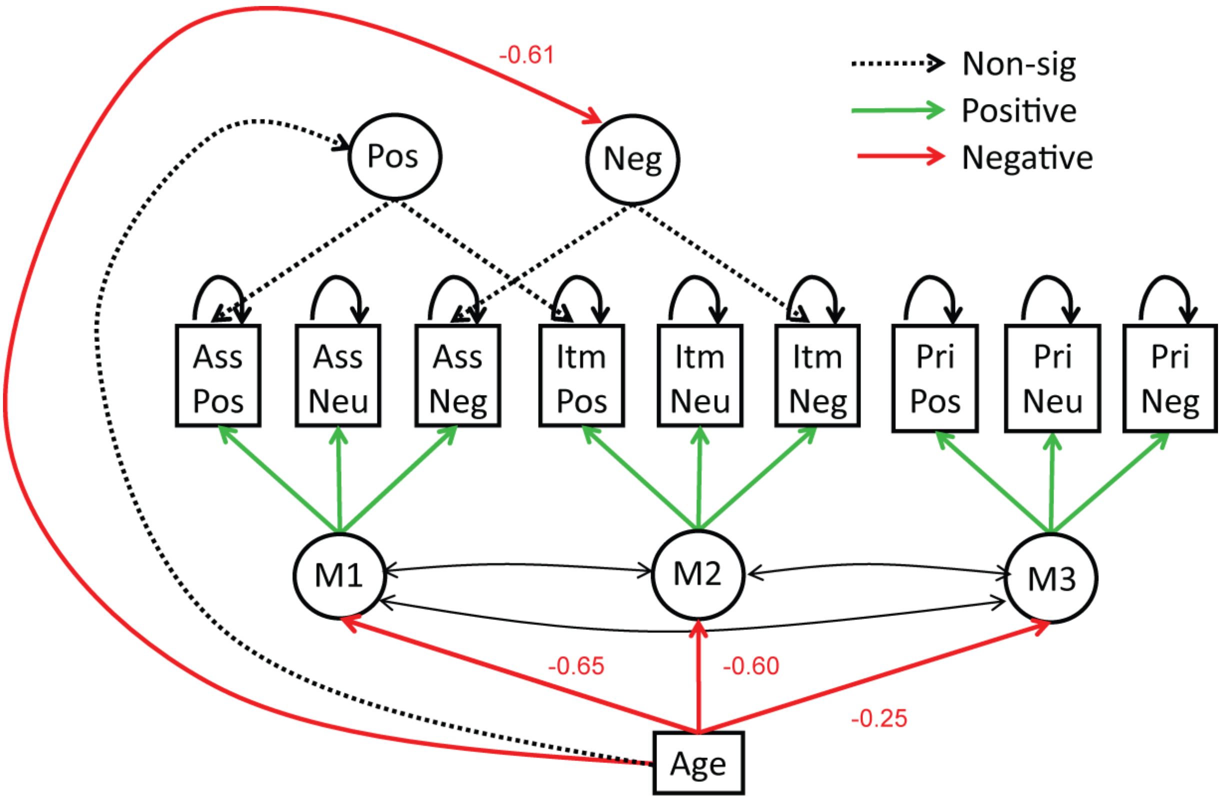

Supplementary Figure 3: *Grey Matter Volumes (GMV) of three, a priori Regions of Interest (ROIs) against Age, fitted by linear (Lin) and quadratic (Qud) components, after adjusting for Total Intracranial Volume (TIV) shown in bottom right panel.*

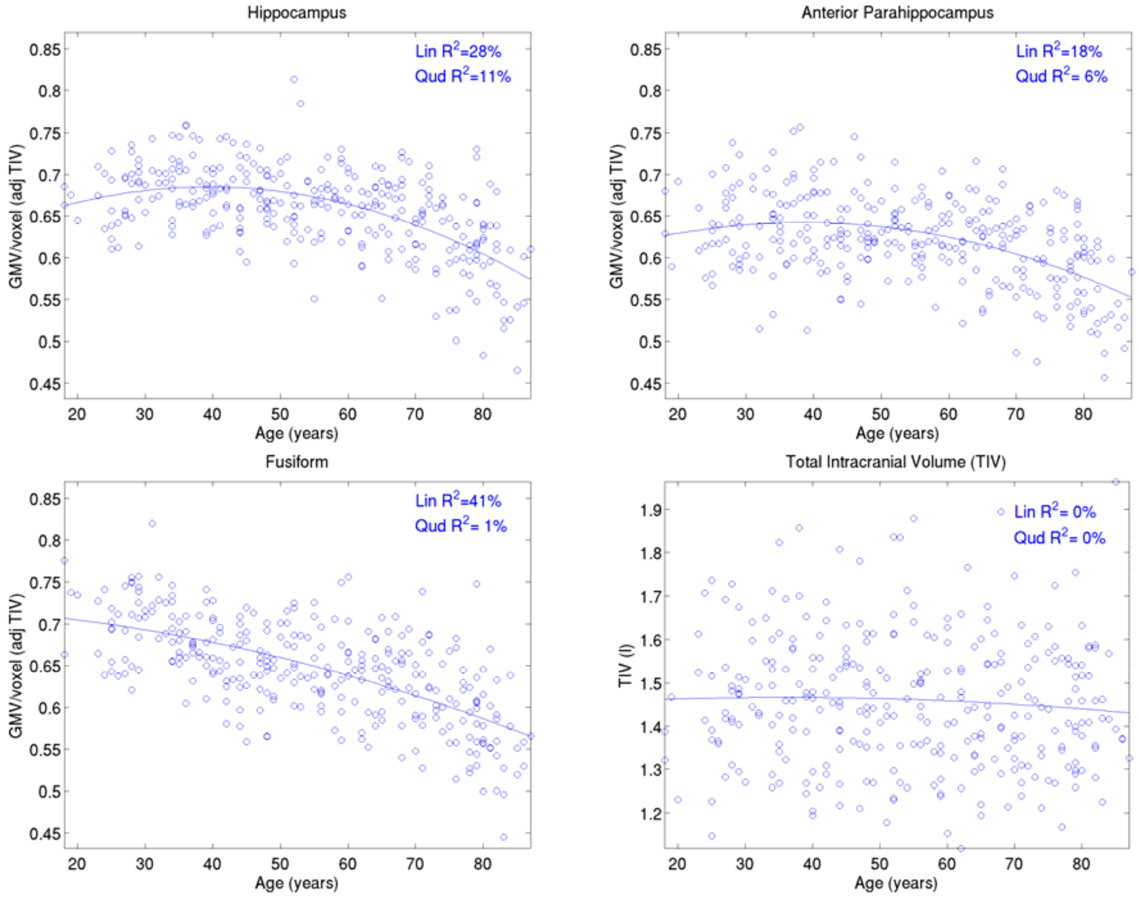

Supplementary Figure 4: *White Matter Index (WMI) of three, a priori Regions of Interest (ROIs) against Age, fitted by linear (Lin) and quadratic (Qud) components.*

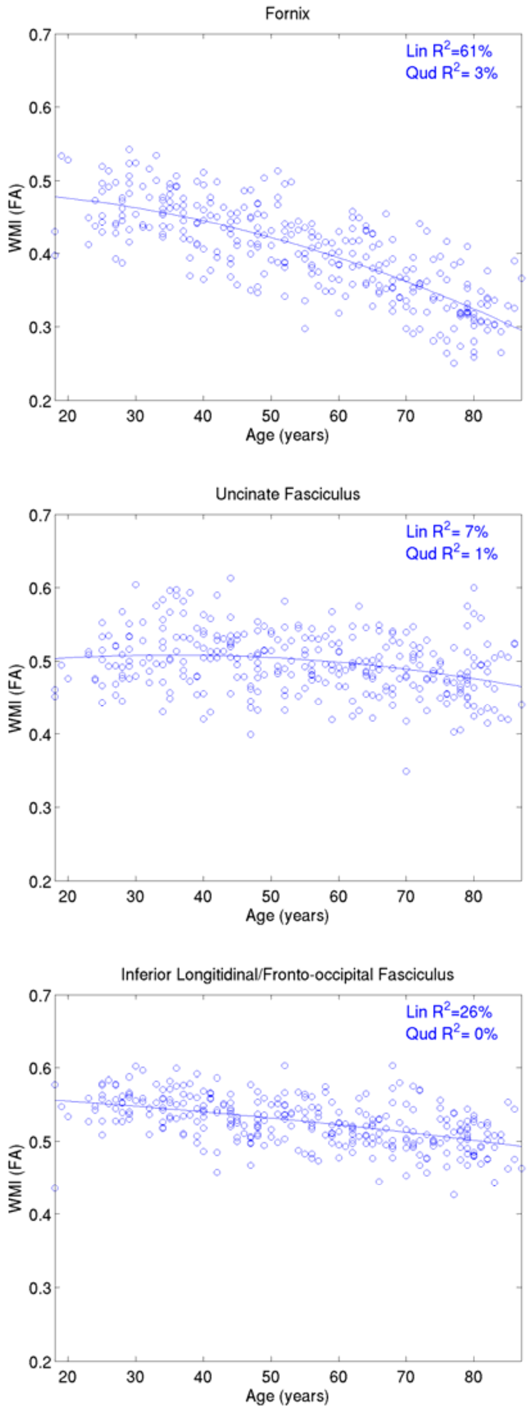

Supplement: Supplementary Information [file srep32527-s1.pdf]
